# To what extent does the Health Professions Admission Test-Ireland predict performance in early undergraduate tests of communication and clinical skills? – An observational cohort study

**DOI:** 10.1186/1472-6920-13-68

**Published:** 2013-05-10

**Authors:** Maureen E Kelly, Daniel Regan, Fidelma Dunne, Patrick Henn, John Newell, Siun O’Flynn

**Affiliations:** 1 Discipline of General Practice, Clinical Science Institute, National University of Ireland, Galway, Ireland; 2 School of Psychology, National University of Ireland, Galway (NUI Galway), Galway, Ireland; 3 The Medical School, National University of Ireland, Galway (NUI Galway), Galway, Ireland; 4 The Medical School, University College Cork (UCC), Cork, Ireland; 5 Health Research Board (HRB) Clinical Research Facility, National University of Ireland, Galway (NUI Galway), Galway, Ireland

**Keywords:** Selection, Medical, Student, Validity, Predictive, HPAT-Ireland, Assessment, Cognitive, Ability

## Abstract

**Background:**

Internationally, tests of general mental ability are used in the selection of medical students. Examples include the Medical College Admission Test, Undergraduate Medicine and Health Sciences Admission Test and the UK Clinical Aptitude Test. The most widely used measure of their efficacy is predictive validity.

A new tool, the Health Professions Admission Test- Ireland (HPAT-Ireland), was introduced in 2009. Traditionally, selection to Irish undergraduate medical schools relied on academic achievement. Since 2009, Irish and EU applicants are selected on a combination of their secondary school academic record (measured predominately by the Leaving Certificate Examination) and HPAT-Ireland score. This is the first study to report on the predictive validity of the HPAT-Ireland for early undergraduate assessments of communication and clinical skills.

**Method:**

Students enrolled at two Irish medical schools in 2009 were followed up for two years. Data collected were gender, HPAT-Ireland total and subsection scores; Leaving Certificate Examination plus HPAT-Ireland combined score, Year 1 Objective Structured Clinical Examination (OSCE) scores (Total score, communication and clinical subtest scores), Year 1 Multiple Choice Questions and Year 2 OSCE and subset scores. We report descriptive statistics, Pearson correlation coefficients and Multiple linear regression models.

**Results:**

Data were available for 312 students. In Year 1 none of the selection criteria were significantly related to student OSCE performance. The Leaving Certificate Examination and Leaving Certificate plus HPAT-Ireland combined scores correlated with MCQ marks.

In Year 2 a series of significant correlations emerged between the HPAT-Ireland and subsections thereof with OSCE Communication *Z*-scores; OSCE Clinical Z-scores; and Total OSCE Z-scores. However on multiple regression only the relationship between Total OSCE Score and the Total HPAT-Ireland score remained significant; albeit the predictive power was modest.

**Conclusion:**

We found that none of our selection criteria strongly predict clinical and communication skills. The HPAT- Ireland appears to measures ability in domains different to those assessed by the Leaving Certificate Examination. While some significant associations did emerge in Year 2 between HPAT Ireland and total OSCE scores further evaluation is required to establish if this pattern continues during the senior years of the medical course.

## Background

The use of tests of general mental ability, including aptitude tests, is widespread in the selection of medical students internationally [1]. Examples include the Medical College Admission Test (MCAT), the BioMedical Admissions Test (BMAT), the Undergraduate Medicine and Health Sciences Admission Test (UMAT) and the UK Clinical Aptitude Test (UKCAT) [2-5]. The hypothesis that establishing medical school applicants’ aptitude at the outset enables one to rank applicants in order of their likelihood to succeed in medicine and become good doctors appears sound on the surface. However the evidence for the effectiveness of such tests, as a selection tool, is mixed and their use is controversial [6].

The most widely used measure of their effectiveness is predictive validity; the ability of the selection tool to predict medical students’ performance in undergraduate assessments. There is consistent (albeit not perfect) evidence for the predictive validity of the MCAT [7,8]. In relation to the UKCAT findings are conflicting. Two studies report no significant correlation between UKCAT scores and medical student performance [9,10]. In a recent follow up study, the authors reported that the UKCAT did not independently predict student performance in clinical course work, whereas prior academic attainment was highly predicitve [11]. Conversely a study from Newcastle University found that the UKCAT significantly predicted exam performance in all but one major exam over two years [12]. Two recently published papers found evidence of little or no predictive validity with respect to the UMAT [13,14]. The modest predictive validity of the BMAT appears to be most related to applicants’ performance in the scientific knowledge section [15,16].

Possible reasons for the variability in reports of predictive validity may stem from comparing research that is limited to single institutions with that from multi-centered studies. Findings reported from single institutions may reflect specific associations with particular curricular or assessment techniques and may not be generalisable to medical schools at large. The reliability and validity of individual medical school assessments, and indeed selection tools may also impact on predictive validity studies. Other potential reasons for variability is the number of students followed up and the duration of follow-up-with larger scale studies, having longer follow up times being more likely to yield valid results.

Although the tests described above all purport to measure aspects of general mental ability there are subtle but important differences between them (See Table 1). One of the most important difference between these tools lies in the domains they assess [1]. For example the MCAT tests both knowledge of physical and biological sciences (termed crystallized intelligence) *and* candidates’ logical reasoning and processing skills (known as fluid intelligence). The BMAT also has a section that tests candidates’ knowledge of science and mathematics. On the other hand the UKCAT and UMAT focus largely on testing candidates’ fluid intelligence in terms of mental processing, reasoning and decision making without testing underlying background knowledge. Whether or not this is fundamental to the differences in predictive abilities has not been fully explored. Knowledge based performance is associated with subsequent success in medical school however in a large meta-analysis Ferguson et al. have established that only approximately 23% of variance in medical school performance can be explained by previous academic performance [17]. Admission tests and aptitude tests therefore are supported because they may measure domains not measured in school exit exams. However their added value to the selection process must be carefully evaluated.

**Table 1:** Features of a variety of general mental ability/aptitude tests compared

**Assessment tool**	**HPAT**	**UMAT**	**UKCAT**	**MCAT**	**BMAT**
Target candidates	Undergraduate medical school applicants – predominately school leavers	Undergraduate medical school applicants – predominately school leavers	Undergraduate medical school applicants – predominately school leavers	Medical School applicants – predominately college students	Undergraduate medical school applicants – predominately school leavers
Type of test	MCQ	MCQ	MCQ	MCQ plus written essay*	MCQ, written answers and written essay
Duration	2 hrs 30 mins	2 hrs 45 mins	2 hrs	4.5 to 5 hours *	2 hr
How administered	Paper based	Paper based	Computer based	Computer based	Paper based
Standard Cost to applicant	€95	€161	€78	€181	€50
No of participating schools	5	14	26	Required by almost all medical schools in North America	6
Year it was first used	2009	First used in 1991 at Newcastle University, Australia with expansion to other institutions in 1997/98	2006	Earliest versions commenced in 1946 and have evolved over time. Current format exists since 1992 with some minor adjustments since	2003
Subsections of test	1. Logical Reasoning & Problem Solving	1. Logical Reasoning & Problem Solving	1. Verbal reasoning	1. Verbal Reasoning Skills	1: Aptitude and Skills
	2. Interpersonal Understanding	2. Understanding People	2. Quantitative reasoning	2. Physical Sciences – chemistry, physics and data interpretation	2: Scientific Knowledge and Application
	3. Non-Verbal Reasoning	3. Non-verbal Reasoning	3. Abstract reasoning	3. Biological Sciences – biology and organic chemistry	3: Writing Task
			4. Decision analysis	* The writing sample section will be removed in 2013 thus shortening the test	

*HPAT*-Health Professions Admission Test Ireland. *UMAT*-Undergraduate Medicine and Health Sciences Admission Test. *UKCAT*- UK Clinical Aptitude Test. *MCAT*- Medical College Admission Test. BMAT-BioMedical Admissions Test.

The Ottawa Consensus Statement on assessment for the selection of health care professions and specialty training strongly recommends that further research and evidence, coupled with an examination of supporting theoretical philosophies is conducted to fully inform the international debate on selection [6].

A new tool, the Health Professions Admission Test- Ireland (HPAT-Ireland), was introduced in 2009 [18]. The main impetus for its introduction was the publication of a Government initiated report which recommended that medical student selection, in Ireland, should no longer be based on academic grades alone. The report acknowledged the increasing use of specialized admission tests which recognize the importance of factors other than academic achievement in the development of a doctor [19]. A key motivator for this recommendation was a sense of social responsibility for widening access to medicine. Candidates from socioeconomically disadvantaged backgrounds are under represented in Irish medical schools; accounting for less than 4% of all applicants [20].

The HPAT-Ireland is designed and independently delivered by the Australian Council for Educational Research-ACER [21]. ACER, a not-for-profit organization specialising in educational decision making, also designs the UMAT exam used by over a dozen institutions in Australia and New Zealand. Information on the development of HPAT-Ireland test items and domains, in particular how these domains are blueprinted against the domains of professional competencies, is not readily available.

The HPAT-Ireland is a multiple choice test. In terms of intelligences tested it largely focuses on fluid intelligence. There are three sections. According to the test designers they measure the following abilities: Section 1: Logical reasoning and problem solving consists of 44 multiple choice questions based on a passage of text or a diagram presenting certain information. Applicants are required to analyse and logically reason through the information presented. Section 2: Interpersonal Understanding consists of 36 multiple choice questions based on a scenario representing specific interpersonal situations. Applicants have to identify, understand, and, where necessary, infer the thoughts, feelings, behaviour and/or intentions of the people represented in the situations. Section 3: Non-Verbal Reasoning consists of 30 multiple choice questions based on recognition of patterns and sequences of shapes. The questions test the applicant’s ability to reason in the abstract and solve problems in non-verbal contexts.

Since the introduction of the HPAT-Ireland, undergraduate medical school places are now offered to Irish and EU school leavers based on a combination of second level school academic achievement (predominately measured by the state run school exit exam the “Leaving Certificate Examination”- LCE) and the applicant’s performance on the HPAT-Ireland (see Additional file 1 for full explanation of selection criteria). Applicants from outside of the EU undergo separate selection processes, outside of the scope of this study.

The National Research Group Evaluating Revised Entry Mechanisms to Medicine is a consortium of medical educators, researchers and statisticians who meet under the auspices of the Council of Deans of the Medical Faculties of Ireland. This group is currently examining the relationship between medical students’ selection scores and their performance on undergraduate cognitive tests. A preliminary report is available but final reports from this work will be available when the initial cohort has completed the five year undergraduate cycle and will be essential to the validation of these selection tools [20].

The focus of this study however is the relationship between student scores in the selection tools and subsequent performance on tests of communication and clinical skills. It is intended that this study will compliment findings from the National Research Group Evaluating Revised Entry Mechanisms to Medicine group and lead to a fuller picture of the utility of these selection tools. Communication and clinical skills are at the heart of sound medical practice. They are cited as two of the eight key domains of good professional practice by the Irish Medical Council [22]. According to the CanMEDS framework communication skills are an essential ability that physicians need for optimal patient care [23]. The corollary is also evident. A breakdown of complaints to the Irish Medical Council reveals that communication problems rank in the top three categories of all complaints received from the public [24]. A similar pattern exists internationally; a survey of three separate American State Medical Boards reported that unprofessional behaviour accounted for 92% of all violations [25]. Sui and Reiter contend that the tradition of demanding high levels of academic excellence for selection to medicine has resulted in limiting the number of complaints in terms of cognitive issues. The new challenge is to identify and include selection tools that screen for other important non-cognitive attributes such as communication skills and professionalism [26]. In modern day curricula, communication and clinical skills are introduced early and built up in a spiral fashion throughout the medical course. A selection tool that could predict strengths in these areas would make a valid contribution to the selection process.

Therefore the aim of this research was specifically to establish whether a relationship existed between student scores on the HPAT-Ireland (including subsections thereof) and the Leaving Certificate Examination and subsequent performance on tests of communication and clinical skills in the early undergraduate years.

## Methods

This study was conducted across two medical schools; National University of Ireland Galway (NUI Galway) and University College Cork (UCC). The competencies of communication and clinical skills are taught at comparable levels throughout the undergraduate courses. At both institutions Objective Structured Clinical Examinations (OSCE) were conducted at the end of Year 1 and Year 2 to assess clinical and communication skills.

### Sample

The sample comprised all students who were enrolled, in their first year of study, at the medical schools of NUI Galway and UCC in the academic year 2009. At NUI Galway, students are either enrolled in Foundation Year (GFY) or First Year Medicine (GMed1) depending on their science subjects grades in the LCE. All students were followed up for two years. Undergraduate examination results for the year of intake, Year 1 (academic year 2009–2010) and the following, Year 2 (academic year 2010–2011) were examined and their association with the selection criteria of LCE and Health Professions Admission Test (HPAT-Ireland) determined.

### Data

ACER and the Central Applications Office provided the HPAT-Ireland and LCE data. The respective medical schools provided the undergraduate examination results. Written consent to use HPAT-Ireland data was given by all the applicants at the time of sitting the HPAT-Ireland. Ethical approval was granted by the Research Ethics Committee, NUI Galway and tabled in UCC. A linked anonymised data base was used for the study. Only the data enterer and a senior academic administrator had access to the link.

The following data were collated: gender, HPAT-Ireland total and subsection scores (Section 1, 2 and 3); LCE, LCE/HPAT-Ireland combined score, Year 1 structured clinical examination (OSCE) scores (Total and subtests (i.e., communication and clinical components), Year 1 Clinical MCQ (total scores only); and Year 2 OSCE (Total and subtest scores).

The LCE adjusted and LCE/HPAT-Ireland combined scores are based on agreed national selection criteria [27] (See Additional file 1). The minimum entry points for medicine (comprising LCE adjusted plus HPAT-Ireland score) in the two medical schools for 2009 were: UCC 715, NUI Galway 712.

The OSCE is designed to test communication and clinical skill performance and competence [28]. The stations in this study assessed a range of skills including diagnosis, history taking, medical procedures and interpretation of results. The score sheets at each medical school allowed for the communication and clinical scores to be extracted from each OSCE station total score. Three outcome OSCE variables were computed (Communication, Clinical and Total) for the samples Galway Year 1 (GY1), Galway Year 2 (GY2), Cork Year 1 (CY1) and Cork Year 2 (CY2). Similarly Multiple Choice Examination (MCQ) outcome scores from clinical modules were extracted to reflect communication and clinical attributes of students. (See Additional file 2 for further details).

While extraction of examination scores was conducted identically in both universities, the OSCE stations were designed and marked differently and so were re-coded as Z-scores (describing each score in terms of its relationship to the class mean score). For GY1, single scores were re-coded for: OSCE Communication (GMed1 and GFY), OSCE Clinical (GMed1 only), Total OSCE (GMed1 only) and finally Multiple Choice Questionnaire (MCQ) which included Communication and Clinical components (GMed1 only). For CY1, single scores were re-coded for: OSCE Communication, OSCE Clinical, Total OSCE, and MCQ. For Y2 at both Galway (minus GFY) and Cork, single scores were re-coded for: OSCE Communication, OSCE Clinical, and Total OSCE. The OSCE stations had both communication and clinical skill components.

Data were analysed using SPSS 17.0 for Windows (SPSS, Inc., Chicago, IL, USA). Descriptive statistics; mean, standard deviation (SD) and median were used to describe continuous variables, and frequencies and percentages to describe categorical variables.

There was no evidence against normality for the continuous explanatory (i.e. HPAT-Ireland and LCE scores) and response variables (i.e. OSCE results) and all were compared between groups (e.g., gender, Foundation Year vs. Med1), using two sample t-tests. The Pearson correlation coefficient was deemed adequate to describe the degree of linear relationship between continuous explanatory and response variables. As outlined in a previous, similar study, limits for correlation coefficients of ≥ 0.20 or ≤ − 0.20 were set as a priori criteria for practical significance [9]. Multiple linear regression models were used to identify significant predictors of the OSCE response variables. Variable selection techniques and the magnitude of the variance inflation factor were used to adjust for multicollinearity due to the correlation between the HPAT-Ireland predictors. A significance level of *p* <0.05 was required for a variable to be included in a model. Given that the percent of missing data varied for each explanatory variable, multiple imputation, using chained equations, was used to impute missing data in order to check the sensitivity of missing data to the identification of significant predictors.

## Results

### Demographics

The total sample was 324 (National University of Ireland, Galway, *n* = 193 [1^st^ Med., *n* = 133; Foundation Year (FY), *n* = 60]; University College Cork, *n* = 131). Of this sample, 46% were male (*n* = 150), and 54% were female (*n* = 174). There was no appreciable difference in gender between the two universities (i.e., % Male: Female, 47: 53 and 45: 55, NUI Galway and UCC respectively). The majority of the sample comprised Irish nationals (83%, n = 269). Age was not ascertained; however, given the typical profile of first year medical students at NUI Galway and UCC, it is anticipated that most participants were between the ages of 18 and 21. A total of 131 students (42%) sat neither the HPAT-Ireland nor the LCE in 2009 (largely comprising non-EU entrants who are selected via a separate process, but also those re-sitting exams or who had deferred entry). Twelve participants were selected via a number of special access routes to study medicine and were excluded from further analysis; leaving a final sample of 312.

*Descriptive statistics* for Years 1 and Year 2 outcome variables are outlined in Table 2.

**Table 2:** Descriptive statistics for the variables of interest and outcome measures

**Variable**	**N**	**M (SD)**	**Median**
Leaving Certificate Examination (LCE)	177	567.57 (21.17)	565.0
Combined LCE/HPAT- Ireland	177	728.36 (14.47)	724.0
HPAT- Ireland Section 1: Logical Reasoning and problem solving (Max = 100)	181	58.82 (7.67)	58.0
HPAT- Ireland Section 2: Interpersonal understanding (Max = 100)	181	56.91 (7.27)	58.0
HPAT- Ireland Section 3: Non-verbal reasoning (Max = 100)	181	60.61 (9.61)	60.0
Total HPAT- Ireland (Max = 300)	181	176.20 (14.38)	174.0
OSCE Year 1Communication *Z*-scores	277	.002 (1.00)	.62
OSCE Year 1 Clinical *Z*-scores	217	-.01 (.99)	.97
OSCE Year 1 – Total OSCE Score	216	- .02 (1.82)	- .05
OSCE Year 2 Communication *Z*-scores	215	.01 (1.00)	.008
OSCE Year 2 Clinical *Z*-scores	210	-.003 (1.01)	.01
OSCE Year 2 – Total OSCE Score	208	.03 (1.85)	-.002
Multiple choice examination	197	.002 (.99)	-.05

Note. *SD* = Standard deviation; *HPAT- Ireland* = Health Professions Admissions Test Ireland; *OSCE* = Objective Structured Clinical Examination.

### Year 1 Group comparisons

A series of two sample t-tests, using a Bonferroni adjustment for multiple testing, were carried out to examine potential differences amongst the students in terms of gender and year of entry to programme. NUI Galway students who entered directly from secondary level schooling into 1^st^ year medicine (*n* = 39), were compared with those entering Foundation Year (*n* = 53) on the variables of interest and the outcome measures (i.e., selection criteria, and medical school examinations). No significant differences were observed on any of these measures, with the exception of isolated differences in HPAT-Ireland Section 3 performances. Therefore all Galway medical students were treated as a single sample.

Further Bonferroni adjusted two sample t-tests revealed that the average score for males was significantly higher, than the average score for females on HPAT- Ireland Sections 1, *t* (179) = 3.51, *p* < .001, *d* = .52, and HPAT-Ireland Section 3, *t* (179) = 3.40, *p* < .001, *d* = .50, but not on the HPAT- Ireland total score. Due to the small numbers in the gender groups (males who undertook HPAT- Ireland and completed Year 1 examinations n = 46, females n = 61) and lack of gender difference on Total HPAT- Ireland performance, analyses were undertaken for the entire sample ^i, ii^.

### Year 2 Group comparisons

A similar series of Bonferroni adjusted two sample comparisons were conducted for Year 2 (i.e. gender and year of entry to programme). There were no significant differences between the groups. Students were therefore treated as a unified sample across all further analyses.

### Correlations

Table 3 shows the correlations between the Communication and Clinical OSCE marks, for Years 1 and 2 respectively. Table 4 shows the correlations between the selection criteria and student performance on the OSCE and the MCQ represented by Z-scores ^iii^. Results for Year 1 are presented below the diagonal and for Year 2 above the diagonal.

**Table 3:** Correlation between communication and clinical elements of OSCE (Galway and Cork – Years 1 and 2)

**Year 1 variables**	**Galway Yr 1 Clinical**	**Cork Yr 1 Clinical**	**Year 2 variables**	**Galway Yr 2-Clinical**	**Cork Yr 2-Clinical**
Galway Yr 1 Communication: Body Mass Index	.32**		Galway Yr 2- Communication- Chest Pain	.20*	
Galway Yr 1 Communication: Vitals	.42**		Galway Yr 2- Communication - Eye exam	.39**	
Galway Yr 1 Communication: Blood Pressure	.55**		Galway Yr 2 Communication -Gastrointestinal	.55**	
Galway Yr 1 Communication: Urinalysis	.45**		Galway Yr 2 Communication - Joint exam	.55**	
Cork Yr 1 Communication: First Aid		.36**	Cork Yr 2 Communication - Neurology History		.73**
Cork Yr 1 Communication: Clinical Anatomy		.45**	Cork Yr 2 Communication – Cardiology		.65**
Cork Yr 1Communication: Respiratory		.78**	Cork Yr 2 Communication - Neurological Lower Limb Examination		.43**
Cork Yr 1 Communication: Abdominal examination		.68**	Cork Yr 2 Communication - Neurological Cranial Nerve Examination		.49**
			Cork Yr 2 - Communication - Cardiovascular exam		.71**
			Cork Yr 2 - Communication - Respiratory exam		.65**

* P<.01, ** P<.05.

**Table 4:** Correlations between selection criteria and outcome measures (Year 1 below diagonal; Year 2 above diagonal)

**Variable**	**HPAT-Ireland 1**	**HPAT-Ireland 2**	**HPAT-Ireland 3**	**Total HPAT-Ireland**	**LCE**	**LCE + HPAT-Ireland**	**OSCE Comm**	**OSCE Clin**	**OSCE Total**	**1MCQ Total**
**HPAT- Ireland 1**	-						.03	.04	.05	n/a
**HPAT-Ireland 2**	-.05	-					.27**	.15	.23*	n/a
**HPAT-Ireland 3**	.29**	-.23**	-				.19	.16	.20	n/a
**Total HPAT-Ireland**	.70**	.32**	.70**	-			.29**	.21*	.28**	n/a
**LCE**	-.12	-.16*	-.06	-.18*	-		-.13	.12	.02	n/a
**LCE + HPAT-Ireland**	.59**	.28**	.61**	.85**	.31**	-	.17	.24*	.24*	n/a
**OSCE Comm**	.-.03	.14	.02	.07	-.03	.04	-			n/a
**OSCE Clin**	.06	-.002	.13	.13	.04	.14	.66**	-		n/a
**OSCE Total**	.10	.07	.07	.18	.02	.18	.91**	.91**	-	n/a
**MCQ Total**	.002	.04	.09	.09	.32**	.27**	.24**	.30**	.30**	-

*HPAT-Ireland* = Health professions admissions test; *LCE* = Leaving certificate examination; *OSCE* = Objective Structured Clinical Examination; *MCQ* = Multiple choice questions; Total = Total scores (i.e., Communication and Clinical elements combined); n/a = not applicable ie there is no result for this test in the corresponding year. * P<.01, ** P<.05.

Correlations between the selection criteria and outcome measures were undertaken for the entire sample.

In Year 1 none of the selection criteria were significantly related to Total OSCE scores. Neither were they related to either OSCE Communication or OSCE Clinical scores. The LCE and LCE/HPAT- Ireland scores were however, positively associated, with MCQ marks (r = .32 & .27 respectively, *p* values all < .01).

In Year 2 moderate, significant associations emerged between HPAT- Ireland 2 and Total HPAT- Ireland, and OSCE Communication *Z*-scores (*r* = .27, .29 respectively; all *p* values < .01). Total HPAT- Ireland and LCE/HPAT- Ireland were significantly correlated (*r* = .21 & .24 respectively; all *p* values < .05) with OSCE Clinical Z-scores. Finally HPAT- Ireland 2, Total HPAT- Ireland, and LCE/HPAT- Ireland were all significantly correlated with Total OSCE Z-scores (*r* = .23, .28, .24 respectively; *p* < .05, .01 & .05 respectively).

### Multiple regression analysis

**Figure 1:**
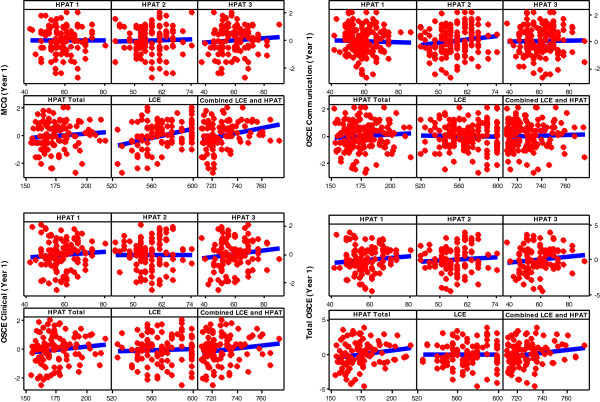
Scatter plot of Year 1 results versus selection criteria.

For the outcome measure Year 1 MCQ score the LCE explanatory variable was identified as the single significant predictor (b = 0.02, p = 0.001, 95% CI 0.007 to 0.024) with an adjusted *R*^2^ of 0.09 suggesting a positive, predictive association between LCE scores and Year 1 MCQ. See Figure 1.

No significant predictors were identified for the OSCE Communication and OSCE Clinical variables at Year 1. For the Total OSCE response, no explanatory variables were deemed useful for inclusion. However the HPAT- Ireland and LCE combined explanatory variables achieved borderline significance (p = 0.06). These results suggest that, based on the sample provided, none of the selection criteria currently used in the Irish system, are predictive of Total OSCE scores in Year 1.

**Figure 2:**
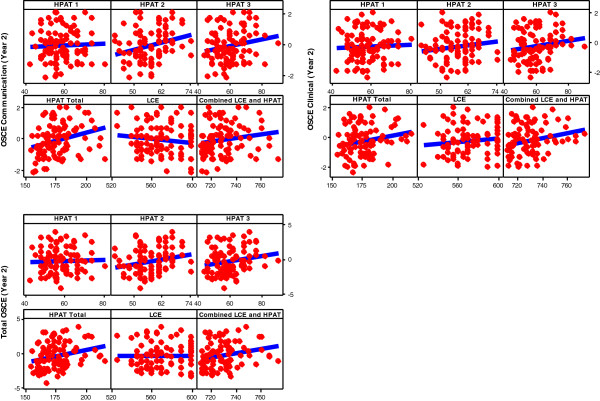
Scatter plot of Year 2 results versus selection criteria.

No significant predictors were identified for the separate OSCE Communication and Clinical response variables at Year 2 response. However, when considering the Total OSCE Year 2 response, Total HPAT- Ireland (b = 0.04, *p* =0.008, 95% CI 0.01 to 0.07), was identified as a significant predictor with a model R^2^ adjusted of 0.07. This suggests that, based on this sample, higher scores on Total HPAT- Ireland scores are related to higher marks on the Year 2 OSCE score however the predictive power is moderate^v^. See Figure 2.

See Additional file 3 for Result Section Notes.

## Discussion

This is the first paper to report on a prospective study establishing the predictive validity of the HPAT- Ireland. We conducted a two year follow up of the first cohort of students, selected to two different medical schools, by the LCE and HPAT- Ireland combined. We examined the relationship between applicant performance on the selection tools, and subsections thereof, and subsequent performance on undergraduate tests of communication and clinical skills.

According to Patterson and Ferguson [1] in criterion related validity studies, such as this one, it is unusual to obtain validity coefficients greater than r =0.5. Values in the range of r = 0.2 to r = 0.29 bracket can be described as low from a practical viewpoint albeit they may reach statistical significance [10]. In a large BEME systematic review of the predictive values of measures obtained in medical school and later performance in medical practice correlations up to and including r = 0.37 were reported as low [29]. Whereas Julian in an analysis of the predictive validity of the MCAT deems values above r = 0.4 or higher as indicative of a fairly strong relationship [8]. When reporting predictive validity studies therefore, it is desirable that correlation coefficients reach at least 0.30 to be considered meaningful [30].

Our first year correlation findings are unremarkable apart from the finding that the LCE and the LCE/HPAT- Ireland correlated with performance in a clinical MCQ. This relationship is to be expected given that both the LCE and the MCQ test in the knowledge domain. This relationship was moderate (*r* = .32 & .27 respectively) and on regression testing only the LCE remained predictive. This is consistent with observations that although prior academic achievement is one of the best predictors of undergraduate medical student performance the majority of the variance in medical student performance lies outside of the influence of this domain [17].

In Year 2 a number of correlations emerge between: OSCE Communication Z Score and HPAT- Ireland Section 2 and Total HPAT- Ireland (r = .27&.29 respectively); OSCE Clinical Z Score and Total HPAT- Ireland and LCE/HPAT- Ireland (r = .21&.24 respectively); and finally between Total OSCE Z Score and HPAT- Ireland 2, Total HPAT- Ireland and LCE/HPAT- Ireland (r = .23, .28 &.24 respectively). However although these correlations reach significance, they are at best moderate. Further analysis, using multiple regression, did not robustly support these correlations, with only Total HPAT- Ireland being somewhat predictive of the Total OSCE Year 2 Z Score.

Specific attention was focussed on correlations between HPAT- Ireland 2 and OSCE Communication Skills sub-scores as this section of HPAT- Ireland purports to assess interpersonal skills. While we did find a correlation, it only emerged in Year 2 and the strength of this relationship was somewhat disappointing. In terms of Clinical Skills sub-scores, our data does not demonstrate a firm relationship with HPAT- Ireland either. Whilst recognising that performance in summative assessment is influenced by a host of variables [17], meaningful correlations between entry criteria and subsequent clinical performance in test conditions would be expected. Indeed for many this is the only added value and justification in the use of adjunct admission tests [31,32]. It is possible however that stronger correlations may emerge as the course progresses and the complexity of clinical assessments increases.

In terms of any evidence of incremental validity (the increase in predictive power by the addition of another selection tool) [1], the data in Table 4 suggest that there may be a possible gain in validity resulting from the addition of the HPAT to the existing selection process. However serial cohort data needs to be analysed to demonstrate this conclusively and multiple regression, at least in Year 1, undermines this observation.

Three types of error are common in validation studies: sampling error due to small sample sizes, poor measurement precision in either the selection tool or the undergraduate assessment tool, and restricted range of scores [1]. Our sample is small by international norms. We attempted to off set this by following up the cohort for two years. Assessment practices at both schools were not identical; and every attempt has been made in the analysis to account for this variance. There is a scarcity of published data on the development and reliability of the HPAT- Ireland. Although it is our understanding that Medical Schools are provided with confidential annual reports on the performance of HPAT- Ireland, these are not readily available in the public domain. We have not adjusted the data to correct for range restriction in HPAT- Ireland. There is not uniform agreement about whether to routinely correct or not [33]. Any one of these limitations could have reduced the size of the correlation between the selection criteria and undergraduate results observed in our study. It is also possible that the HPAT- Ireland and/or the LCE predict performance outside of the domains we examined.

Two previous publications reported on the HPAT- Ireland [34,35]. However, drawing generalised conclusions from these studies is limited by the fact that in both cases a scaled down, modified version of the HPAT- Ireland was used.

We found that on average males scored significantly higher than females in HPAT-Ireland Section 1 (logical reasoning & problem solving) and HPAT- Ireland Section 3 (non-verbal reasoning). We found no gender difference in our sample in relation to Leaving Certificate or HPAT- Ireland Section 2 scores. This is surprising, as it is well established with respect to the Leaving Certificate that females perform better overall [36]. It may be that our sample size was too small to detect true difference between the genders. We report no gender difference on total HPAT- Ireland score. However further research is required before confident statements can be made about the role of gender in HPAT- Ireland performance. Similar concerns have been raised with respect to the UKCAT [37].

Correlations between the LCE and the total HPAT- Ireland showed a very weak negative relationship (r = −.18). This may reflect that the LCE and the HPAT- Ireland are examining different applicant attributes. A recent study compared the predictive validity of the Undergraduate Medicine and Health Sciences Admission Test (UMAT) and Grade Point Average (GPA) [13]. GPA was found to be a better overall predictor of medical school exam performance than the UMAT, but the UMAT and GPA together were marginally better again. For senior students the UMAT offered no predictive advantage over the GPA, with respect to communication and clinical skills. These findings are of particular relevance as the HPAT-Ireland and the UMAT are both designed by ACER and have comparable subsection domains.

The HPAT-Ireland is one of the latest tests of general mental ability to appear on the selection scene. Its design and item content closely resembles that of the UMAT. The inclusion of this test was controversial with many suggesting that reforms in Ireland represented a missed opportunity to introduce a test which demonstrably added value to the selection process [38,39]. For example the incorporation of situational judgment tests looks promising and has the potential to improve the utility of tests of general mental ability as a selection tool [40]. The real benefit of this class of tests is their ability to be taken by large numbers of candidates with minimal cost in terms of finance and medical school faculty time. However the challenge for test designers is to continually improve the design of such tests so that the domains that they assess help us to rank medical school applicants in a meaningful way.

## Conclusions

At present it appears that none of the entry and selection criteria used in the Irish system strongly predict clinical and communication skills performance in the early stages of the course. Some correlations emerge between total HPAT –Ireland scores, HPAT section 2 (measuring interpersonal understanding) and subsequent OSCE performance but correlations are weak to moderate. Further analysis is necessary and is ongoing. Any additional selection test must add value to the selection process in general and it is desirable that such tests enhance of the ability of schools to select candidates with an aptitude for clinical and communication skills. While the HPAT- Ireland appears to measures ability in domains different to those assessed by the LCE it remains to be conclusively established whether this correlates robustly with subsequent medical school performance. This cohort will be followed up for their remaining years in medical school and further evaluations will be conducted to establish if this pattern continues into the senior years of the course.

## Abbreviations

ACER: Australian Council for Educational Research; BMAT: BioMedical Admissions Test; CY1: University College Cork, First Year Medicine; CY2: University College Cork, Second Year Medicine; EU: European Union; GFY: National University of Ireland, Galway Foundation Year; GMed1: National University of Ireland, Galway, First Year Medicine; GY2: National University of Ireland, Galway, Second Year Medicine; HPAT: Ireland Health Professions Admission Test-Ireland; HRB: Health Research Board; LCE: Leaving Certificate Examination; MCAT: Medical College Admission Test; MCQ: Multiple Choice Questions; OSCE: Objective Structured Clinical Examination; NUI Galway: National University of Ireland, Galway; UCC: University College Cork; UKCAT: UK Clinical Aptitude Test; UMAT: Undergraduate Medicine and Health Sciences Admission Test.

## Competing interests

FD and SO’F are members of the National Research Group Evaluating Entry and Selection to Medical Schools. This group comprises Deans and Heads of Medical Schools and is broadly evaluating the impact of the changes to Irish medical student selection criteria.

## Authors’ contributions

All authors made substantial contributions to the conception and design of the study, interpretation of findings and write up. MK and FD provided NUI Galway data. PH and SOF provided UCC data. SOF provided HPAT-Ireland and LCE data. DR inputted raw data and conducted statistical analysis. JN advised on study design, data analysis and results interpretation. MK was principal investigator for the study. She wrote the first draft of the manuscript, with DR’s input for the results section. All authors contributed to its revision and approval of final submission.

## Pre-publication history

The pre-publication history for this paper can be accessed here:

http://www.biomedcentral.com/1472-6920/13/68/prepub

## Supplementary Material

Additional file 1 Agreed National Selection Criteria for Undergraduate Medical Schools in Ireland. Click here for file

Additional file 2 OSCE details. Click here for file

Additional file 3 Result Section Notes. Click here for file
