# Supplementary material for: To what extent does the Health Professions Admission Test-Ireland predict performance in early undergraduate tests of communication and clinical skills? – An observational cohort study
Source: BMC Med Educ. 2013 May 10;13:68. doi: 10.1186/1472-6920-13-68 (PMC3667098; doi:10.1186/1472-6920-13-68)
Supplement: Additional file 1 — Agreed National Selection Criteria for Undergraduate Medical Schools in Ireland. [file 1472-6920-13-68-S1.docx]

**Additional File 1: Agreed National Selection Criteria for Undergraduate Medical Schools in Ireland**

The Leaving Certificate Examination adjusted and Leaving Certificate Examination/HPAT-Ireland combined scores are based on the agreed national selection criteria for undergraduate medical schools in Ireland. These criteria dictate the relative weighting of the LCE and the HPAT-Ireland. Candidates must achieve a Leaving Certificate performance of at least 480 points, where an A1 or score of greater than 90% represents 100 points, an A2 or score of 85-89 represent 90 points, B1 or score of 80-84 represents 85 points.

In 2009 the maximum possible combined score was 860; 560 attributable to the LCE and 300 to the HPAT-Ireland [1]. The “LCE adjusted” is a moderated LCE score after a sliding scale has been applied to the raw LCE score. Above a cut-off of 550 points, every additional 5 points achieved on the LCE is awarded 1 point; so that a raw LCE score of 600 becomes 560 after adjustment.

**Reference:**

1. Central Applications Office 2012. **Admission Data 2009** http://www2.cao.ie/points/lvl8_09.pdf (cited 20/11/2012)
