# Supplementary material for: To what extent does the Health Professions Admission Test-Ireland predict performance in early undergraduate tests of communication and clinical skills? – An observational cohort study
Source: BMC Med Educ. 2013 May 10;13:68. doi: 10.1186/1472-6920-13-68 (PMC3667098; doi:10.1186/1472-6920-13-68)
Supplement: Additional file 2 — OSCE details. [file 1472-6920-13-68-S2.doc]

**Additional File 2: OSCE details**

OSCE reliability was calculated using Chronbach Alphas coefficients. Chronbach Alphas are reported for Total OSCE, OSCE Clinical and OSCE Communication station items.

**GY1 Total OSCE = .49 (95% CI=.32 - .62)**

GY1 OSCE Clinical = .41 (95% CI = .21 - .56)

GY1 OSCE Communication = .59 (95% CI =.45 - .70)

**GY2 Total OSCE = .67 (95% CI =.56 - .76)**GY2 OSCE Clinical = .70 (95% CI = 61 - .77)

GY2 Comm =  .78 (95% CI = .72 - .83)

**CY1 OSCE Total: .55 (95% CI=.39 - .67)**CY1 OSCE Clinical = .80 (95% CI = .75 - .85)

CY1 OSCE Communication = .65 (95% CI = .54 - .74)

**CY2 OSCE Total: .50 (95% CI=.32 - .64)**

CY2 OSCE Clinical = .81 (95% CI = .76 - .86)

CY2 OSCE Communication = .57 (95% CI = .43 - .68)
