# Supplementary material for: To what extent does the Health Professions Admission Test-Ireland predict performance in early undergraduate tests of communication and clinical skills? – An observational cohort study
Source: BMC Med Educ. 2013 May 10;13:68. doi: 10.1186/1472-6920-13-68 (PMC3667098; doi:10.1186/1472-6920-13-68)
Supplement: Additional file 3 — Result Section Notes. [file 1472-6920-13-68-S3.docx]

**Additional File 3 Result Section Notes**

^i^ There was a significant difference in the proportion of those sitting/ not-sitting the LCE and HPAT-Ireland exams between NUI GALWAY (e.g., % HPAT-Ireland: No-HPAT- Ireland: 49:51) and UCC (e.g., % HPAT- Ireland: No-HPAT- Ireland: 73:27).

^ii^ Gender difference on HPAT- Ireland 2 approached significance: *t* (182) = -2.79, *p* = .006, *d* = -.41, with males having lower scores).

^iii^ Some results are connected only with certain parts of the sample. For example, Galway Foundation year had no OSCE clinical station results provided, and therefore the OSCE Clinical results, refer to 1^st^ Med Galway and 1^st^ Med Cork only.

^iv^ ­It should be noted that some researchers suggest a more conservative *r* = .3 minimum threshold for inclusion within regression analyses.

^V^ A sensitivity analysis of the conclusions made to the presence of missing data was performed where each final model was refitted for each imputed data set and the results pooled over the five sets. The same predictors were identified for each analysis suggests that final conclusions are not sensitive to missing data.
